# Partial differential equations modeling of bio-convective sutterby nanofluid flow through paraboloid surface

**DOI:** 10.1038/s41598-023-32902-z

**Published:** 2023-04-15

**Authors:** Muhammad Abdul Basit, Muhammad Imran, Shan Ali Khan, Abdullah Alhushaybari, R. Sadat, Mohamed R. Ali

**Affiliations:** 1 Department of Mathematics, Government College University Faisalabad, Faisalabad, 38000 Pakistan; 2 Department of Mathematics, College of Science, Taif University, P.O. Box 11099, Taif, 21944 Saudi Arabia; 3 Department of Mathematics, Faculty of Engineering, Zagazig University, Zagazig, Egypt; 4 Faculty of Engineering and Technology, Future University in Egypt, New Cairo, 11835 Egypt; 5 Basic Engineering Science Department, Benha Faculty of Engineering, Benha University, Benha, Egypt

**Keywords:** Engineering, Applied mathematics, Computational science

## Abstract

In this research article, the behavior of 2D non-Newtonian Sutterby nanofluid flow over the parabolic surface is discussed. In boundary region of surface buoyancy-driven flow occurred due to considerable temperature differences produced by the reaction happen between Sutterby nanofluid and catalyst at the surface. Free convection which is sighted easily on the parabolic surface is initiated by reaction on the catalyst surface modeled the 1st order activation energy. Applications of parabolic surfaces are upper cover of bullet, car bonnet, and air crafts. Under discussion flow is modelled mathematically by implementing law of conservation of microorganism’s concentration, momentum, mass and heat. The governing equations of the system is of the form of non-linear PDE’s. By the use of similarity transform, the governing PDE`s transformed as non-dimensional ODE’s. The resultant system of non-dimensional ODE’s are numerically solved by built-in function MATLAB package named as ‘bvp4c’. Graphical representation shows the influence of different parameters in the concentration, velocity, microorganisms and temperature profiles of the system. In temperature profile, we examined the impact of thermophoresis coefficient *Nt *(0.1, 0.5, 1.0), Prandtl number *Pr *(2.0, 3.0, 4.0), and Brownian motion variable *Nb *(0.1, 0.3, 0.5). Velocity profile depends on the non-dimensional parameters i.e. (Deborah number *De* & Hartmann number *Ha*) and found that these numbers (*De, Ha*) cause downfall in profile. Furthermore, mass transfer, skin friction, and heat transfer rates are numerically computed. The purpose of the study is to enumerate the significance of parabolic surfaces for the transport of heat and mass through the flow of bio-convective Sutterby nanofluid.

## Introduction

The fluid exists in all places, in layman approach fluid means “liquid” but after consulting the literature we found that there are different types of fluids i.e. (liquids, gases etc.) and it is categorized in different types such as compressible or incompressible, rotational or irrotational, steady or unsteady, viscous or non-viscous. Nanofluids have many applications especially in our daily life like bio-medicine, computer chips, mobile phones, refrigeration, heat exchanger, electronics and in fuel industry as well.

Now the scientist working on nano-fluids which is identified by S.U.S Choi^1^ as it is fluid containing nano-metered particles. He purported that heat transfer through these particles is higher than simple heat transfers and nano-fluid has high thermal conductivity. Sabir et al*.*^2^ purported the effects of thermal radiation on Sutterby fluid moving over an inclined surface. Madhukesh et al*.*^3^ unveiled the concept of radiative and chemically reactive casson nanofluid past through a porous media. Song et al*.*^4^ investigated the flow of Sutterby nano-fluid over stretched cylinder involving microorganisms with Marangoni & Solutal conditions. Different effects (i.e. Brownian & thermophoresis) are also considered and construct mathematical model. For transforming our model into dimensionless form, we introduced suitable parameters. Waqas et al*.*^5^ considered the Sutterby nanofluid flow inside the coaxially rotating stretching disks. Nano-fluids play vital role in transport of heat and mass because these are highly conductive & radiative. By considering motile micro-organisms heat transfer rate becomes more frequent. For future consideration Ramesh et al*.*^6^ disclosed the way for inquiring energy transport in the presence of magnetic field with slip condition. Usman et al*.*^7^ reported the non-Newtonian Sutterby nanofluid flow and heat & mass transport on a curved stretching sheet. Darcy–Forchheimer model is used because of porosity and curved stretching sheet. Gyrotactic microorganisms swimming impact also included in the governing equation of motile microorganism.

Mabood et al*.*^8^ evaluated the result on taking the spinning stretchable disk and considered MHD sutterby fluid over it. Stefan blowing and Fourier and Fick’s both well-known models of heat and mass transport are improved by considering Cattaneo–Christov (CC) model for such type of problems. Sajid et al*.*^9^ investigated the effect of thermal slip boundary on the Sutterby nanofluid flow through a slippery sheet by considering nanoparticles of gold in base fluid. Aldabesh et al*.*^10^ endorsed that the parametric effects change the nature of problems by considering Sutterby nano-fluid involving microorganisms over stretched cylinder. For showing these effects Darcy resistance, thermal radiation and activation energy play important role. By implementing the theories of Fourier & Fick extended problems are studied at stationery point and stretched cylinder. Bouslimi et al*.*^11^ considered the model of Sutterby nanofluid for checking thermal characteristics in this model nanoparticles of copper and sodium alginate used. Faizan et al*.*^12^ emphasized on the important model of Riga plate in which magnets and electrodes are adjusted for the need of electric magnetization. This setup visualizes electromagnetic hydrodynamic behaviour of fluid flow. For industrial and thermal purposes, Sutterby nano-fluid flow through Riga plate gives the wonderful applications. Entropy analysis also done when Riga plate is considered and flow of Sutterby nano-fluid over it. Over Riga plate, for the better flow of mass and heat transport motile microorganisms are considered.

Activation energy is a term that was first introduced by Swedish Svante Arrhenius in 1889 and afterward further investigated by many researchers which is a minimal quantum of energy required to institute a chemical reaction/process. Shah et al*.*^13^ discussed a comparative analysis of electrically radiative Casson nanofluid. Zeeshan et al*.*^14^ investigated special functional fluids called nanofluids are created to maximize heat transfer while minimizing energy loss. For this type of fluids, the particle's Brownian and thermophoresis motion play key roles for the movement of heat. The increase in heat transfer has stimulating impacts on energy dissipation restraint and entropy generation reduction. Non- Newtonian Casson nano-fluid also two-dimensional flow over an outer plane which is horizontal surface of parabola is examined. It was found that the chemical process parameter is what caused the temperature field to rise, and the Casson fluid parameter caused a decrease in the temperature profile. Punith et al*.*^15^ purported that wide variety of fields including coatings, oceanography, suspensions, metallic plates coolants, heat exchangers, biological fluids of moving nature, and melt-spinning, use activation energy on non-Newtonian nanofluids for flow and heat transport. Sohaib et al*.*^16^ revealed that how a tangential hyperbolic nano-fluid flow across an extensible Riga wedge transfers heat and mass in the existence of heat source, stagnation point, and activation energy. Fazal et al*.*^17^ discussed mixed convection MHD Casson nano-material flow with activation energy over the stretchable cylinder. At the surface's edge, the effects of solutal, thermal, and motile density stratifications are also considered. Venkateswarlu et al*.*^18^ emphasized on dissipative flow of hybrid nano-fluid involving Propylene-glycol and water through a sphere by considering presence of heat source and chemical reaction parameters. Benhanifia et al*.*^19^ discussed the flow of Casson nanofluid with mixing through a cylindrical vessel and numerical results computed computationally. Haq et al*.*^20^ investigated in the current analysis, 2D mixed convection radiative nanofluid flow and the non-Darcy porous medium with Chemical Reaction are seen across a wavy slope. Jamshed et al*.*^21^ simulated the Williamson nanofluid involving copper and aluminium oxide nanoparticles in base fluid of methanol flowing through solar collector.

For a long time, people have been aware of the occurrence of 'bioconvection' (Loeffer & Mefferd 1952). In liquid suspensions of swimming micro-organisms, cellular streaming patterns are observed in which bulk fluid motions occur downwards in places where high concentrations of micro-organisms develop and upwards in regions of low concentration. Bagh et al*.*^22^ elaborated how proficient computational method is used to conduct an analysis for changing temperature with bioconvection of self-motivated microorganisms blended in micropolar based nanofluids. Around a point of stagnation over a stretching/shrinking sheet, the total transit of concentration, momentum, and energy occurs. Hassan Waqas et al*.*^23^ purported that the attention of the scientists is on this area because nanoparticles are dynamically used in several bio-medical and engineering domains, such as micro-electronics, cooling, heating process, and chemotherapy. In order to do this, the current conversation is driven by the desire to better understand the melting phenomenon on a revolving wedge and the effects of cross nanofluid conformist Falkner-Skan bio-convection flow. Khan et al*.*^24^ designed the recent mathematical structure to examine results of magnetised viscous nano-fluid flowing with bio-convection effect past various geometries (plate, wedge, and cone) with convective boundary constraints. Imran et al*.*^25^ analyzed the weightage of bioconvection on magnetic ferro-fluid with nano-particle suspension and motile microorganisms is an important area of research because of the variety of applications they have. The magneto-viscous origin of ferro-fluids is directly influenced by magnetic nano-structures, which significantly improves the fluid's viscosity and thermo-physical properties. Hayat et al*.*^26^ investigated Prandtl-Eyring nanomaterial in the presence of gyrotactic microorganisms. Din et al*.*^27^ visualized the Carreau nanofluid flow through a stagnation point under the magnetic effect and tackled system with spectral local technique. Asjad et al*.*^28^ worked on the motion of the nanofluids that idea utilized effectively to limit the enhancement of heat transport caused due to stretched sheets. Rehman et al*.*^29^ studied the convective nanofluid flow through a movable needle and also taken into account soret and dufour effect. Wang et al*.*^30^ focused on a 3D Maxwell bio-convective nano-material liquid flow that is erratic and moving toward a surface which is expanding exponentially underneath the impact of chemical process slip conditions. Madhukesh et al*.*^31^ treated bioconvective nanofluid flow over a Riga plate. Imran et al*.*^32^ discussed the physical characteristics of nanofluid flow with the effect of bio-convection via parabolic of revolution across a parallel surface with gyrotactic motile micro-organisms. Shahzad et al*.*^33^ inquired the heat and mass transport through solar water engine flowing Oldroyd-B nanofluid with aluminium alloy. Awan et al*.*^34^ emphasized on the efficacy of non-linear chemical reaction and thermal radiation on stretching sheets, this paper examines the impact of bio-convection on Williamson nanofluid flow. Ramesh et al*.*^35^ purposed a study of nanoliquid with magnetic field flowing over a porous revolving thin needle.

**Figure 1:**
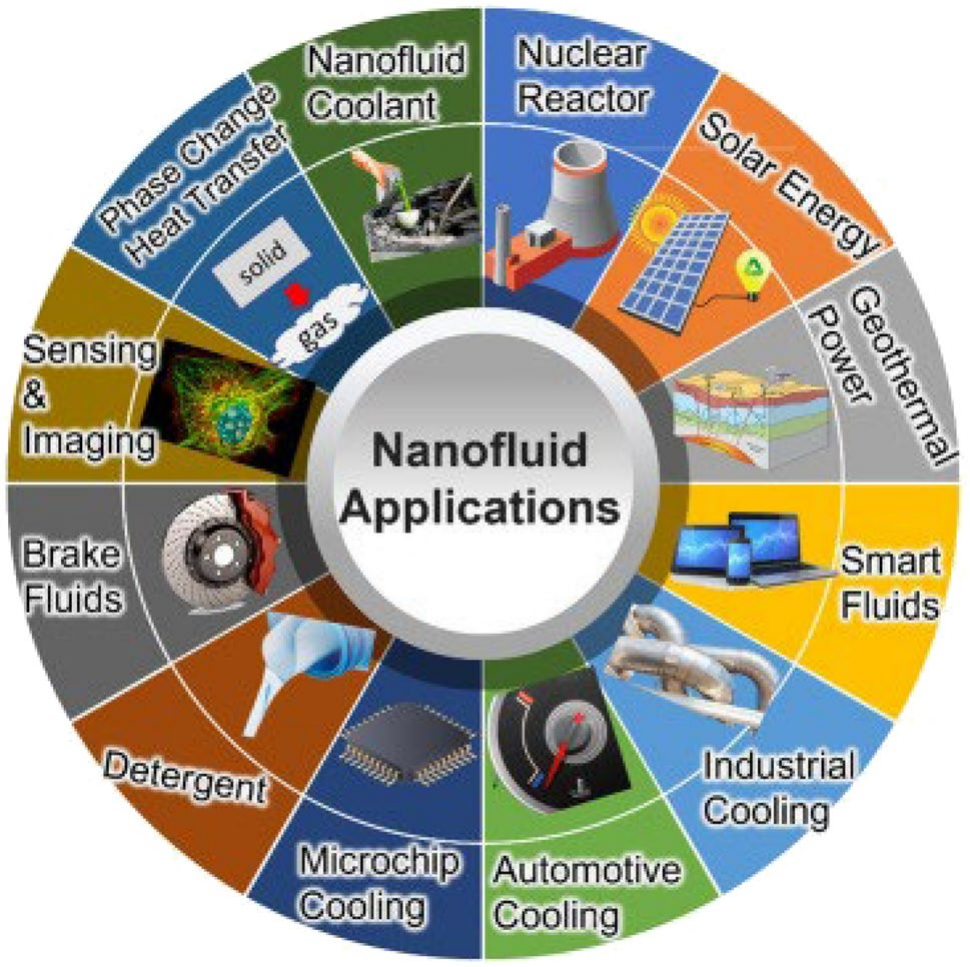
Illustrated physical applications of nano-fluids.

Thermal radiation is a form of energy which emitted from energized body in all directions and moves directly to the point of absorption. Planck`s law and Stefan-Boltzmann law well defined the thermal radiation. Yahaya et al*.*^36^ explored the action of suction on a 2D natural convective flow of unsteady electrical MHD nano-fluid over a linearly permeable stretching sheet, as well as chemical process, Joule heating, viscous dissipation, and thermal radiation. Sobamowo^37^ conducted investigation into the impacts of nano-particles and thermal radiation on the rate of heat transport in Casson nanofluids and free convection flow along a perpendicular surface. Venkata et al*.*^38^ attempted to use the Homotopy analysis method to determine the administering constraints of heat and flow of a nano-fluid throughout a flat surface. Shahzad et al*.*^39^ purported the idea of Sutterby nanofluid past through a sloping sheet in the presence of thermal radiation and magnetic field. Dash et al*.*^40^ discussed thermal radiation with magnetohydrodynamic (MHD) nanofluid over a moving vertical plate causes heat transfer phenomena. Rehman et al*.*^41^ investigated the impacts of thermal radiation and chemical reaction on the flow past of Sutterby nanofluid through a stretchable surface by the utilization of Cattaneo-Christov heat and flux model. Figure 1 represented some important applications of nanofluids in real world.

Various research articles are subjected to Sutterby nanofluid found during the course of literature but in current scenario Sutterby bioconvective nanofluid is considered for the transfer of heat and mass transport involving motile microorganisms over a paraboloid surface. Also considered the impacts of thermal radiation and Chemical reaction on the flow. Main purpose of our work is to show the effect of involvement of motile micro-organisms on heat and mass transfer rate. These types of surfaces are used in industrial systems (i.e. submarine`s and motion of projectile). This study was designed to provide answers to following research questions:

- How heat and mass transfer is conveyed by considering Bio-Convection.
- By adding thermal radiation, is the thermal conductivity increased or not.
- Suitable parameters are introduced to simulate governing equations.
- In graphs and tables, the impacts of dynamic parameters are visualized?
- Novel significance in thermal processes, bio-fuels, heat transfer devices is may be presented by obtained simulation.

## Mathematical structure of problem

**Figure 2:**
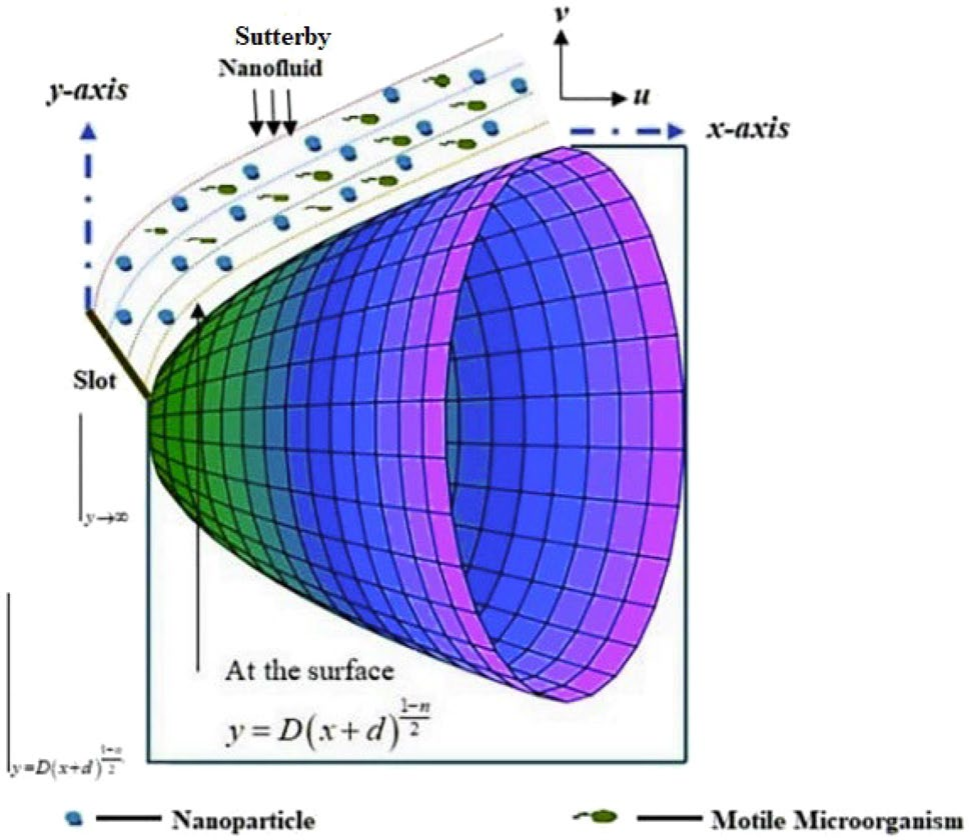
Demonstrates the physical geometry of the problem.

Let us consider a two-dimensional flow of Sutterby nanofluid through a parabolic surface under the influence of magnetic field in Fig. 2. Under consideration surface which is assumed to be paraboloid is considered as solid and non-permeable.

Domain of Sutterby nano-fluid we have taken is $A\left( {x + e} \right)^{{\frac{1 - n}{2}}} \le y \le \infty$. Free stream stretching velocity which is of the form $U_{w} = U(x + e)^{n}$, where n is power index of velocity having domain $0 < n < 1$, stretching parameter *e,* and stretching rate constant is U. flow directions are to be taken x axis as horizontal and y axis as normal directions. Initial point of the flow is at the slot which is a function $y = A\left( {x + e} \right)^{{\frac{1 - n}{2}}}$. From Ref^42^ Sutterby fluid is mathematically represented in the form as

1 $$T = - \rho I + S,$$

and $S = \frac{\mu }{2}\left[ {\frac{1}{\delta \gamma }Sinh^{ - 1} (\delta \gamma )} \right]^{m} A_{1} .$ here $A_{1}$ represents Rivlin Erickson tensor which is computed as $A_{1} = \vec{\nabla }\mathop{V}\limits^{\rightharpoonup} + (\vec{\nabla }\mathop{V}\limits^{\rightharpoonup} )^{t} ,$ $\mu$ represents the fluid`s viscosity, $\gamma$ represents the 2nd invariant strain tensor which is mathematically written as $\gamma = \sqrt {\frac{1}{2}tr(A_{1} )^{2} } ,$ *m* represents power law index, $V = [u(x,y),v(x,y),0]$ represents velocity field, $\delta$ be the material constant. 2nd order approximation of $Sinh^{ - 1}$ function is

2 $$Sinh^{ - 1} (\delta \gamma ) \cong \delta \gamma - \frac{{(\delta \gamma )^{3} }}{6}, \left| {\delta \gamma } \right| \ll 1.$$

From Ref.^43^ mathematical equation that represents 2D heat and mass transport of Sutter by nano-fluid is as:

3 $$\vec{\nabla }.\,\mathop{V}\limits^{\rightharpoonup} = 0,$$

4 $$\rho (\mathop{V}\limits^{\rightharpoonup} .\vec{\nabla })\mathop{V}\limits^{\rightharpoonup} + \vec{\nabla }p - div\,S + J \times B - \rho g_{x} = 0,$$

5 $$(\rho c)_{f} (\mathop{V}\limits^{\rightharpoonup} .\vec{\nabla })T - k\vec{\nabla }^{2} T - (\rho c)_{p} \left[ {D_{B} \vec{\nabla }C.\vec{\nabla }T + \frac{{D_{T} }}{{T_{\infty } }}\vec{\nabla }T.\vec{\nabla }T} \right] = 0,$$

6 $$(\mathop{V}\limits^{\rightharpoonup} .\vec{\nabla })\overline{C} - D_{B} \vec{\nabla }.\vec{\nabla }.\overline{C} - \frac{{D_{T} }}{{T_{\infty } }}\vec{\nabla }.\vec{\nabla }.\overline{T} = 0$$

7 $$\vec{\nabla }.\hat{j} = 0,$$

where $\vec{\nabla }^{2} = \partial^{2} /\partial x^{2} + \partial^{2} /\partial y^{2} ,\,q = \left( {u,v} \right),\,\hat{j} = N\left( {q + \mathop{V}\limits^{\rightharpoonup} } \right) - D_{n} \vec{\nabla }N,\,\,\,\mathop{V}\limits^{\rightharpoonup} = \left( {bW_{c} /\Delta C} \right)\vec{\nabla }C,$ and $Q_{0}$ represents internal heat coefficient.

In above equations different parameters involved here we elaborate these parameters, $D_{B}$, $T$,* p*, $\rho$, *n*, *C*, *B*, $D_{T}$, *J*, and* k* are the Brownian motion diffusivity, temperature field, pressure, density, velocity index of paraboloid surface, concentration field, magnetic field, thermophoretic diffusion coefficient, current density, and thermal conductivity respectively. Component forms of above Eqs. (3–7) are:

7 $$\frac{\partial u}{{\partial x}} + \frac{\partial v}{{\partial y}} = 0,$$

8 $$\begin{aligned} & \rho \left( {u\frac{\partial u}{{\partial x}} + v\frac{\partial u}{{\partial y}}} \right) + \frac{\partial p}{{\partial x}} - \frac{{\partial S_{xx} }}{\partial x} - \frac{{\partial S_{xy} }}{\partial y} + \sigma B_{0}^{2} u + g\left( {\frac{n + 1}{2}} \right) \\ & \left[ {\left( {T - T_{\infty } } \right)\left( {1 - C_{\infty } } \right)\beta \rho_{f} - \left( {\rho_{m} - \rho_{f} } \right)\left( {N - N_{\infty } } \right) - \left( {C - C_{\infty } } \right)\left( {\rho_{p} - \rho_{f} } \right)} \right] = 0, \\ \end{aligned}$$

9 $$\rho \left( {u\frac{\partial u}{{\partial x}} + v\frac{\partial u}{{\partial y}}} \right) + \frac{\partial p}{{\partial y}} - \frac{{\partial S_{yx} }}{\partial x} - \frac{{\partial S_{yy} }}{\partial y} + \sigma B_{0}^{2} v = 0,$$

10 $$\left( {\rho c} \right)_{f} \left( {u\frac{\partial T}{{\partial x}} + v\frac{\partial T}{{\partial y}}} \right) - k\frac{\partial }{\partial y}\left( {\frac{\partial T}{{\partial y}}} \right) - \left( {\rho c} \right)_{p} \left( {D_{B} \frac{\partial T}{{\partial y}}\frac{\partial C}{{\partial y}} + \frac{{D_{T} }}{{T_{\infty } }}\frac{\partial T}{{\partial y}}\frac{\partial T}{{\partial y}}} \right) + \frac{{\partial q_{r} }}{\partial y}\frac{1}{{\left( {\rho c_{p} } \right)}}\, = 0,$$

11 $$u\frac{\partial C}{{\partial x}} + v\frac{\partial C}{{\partial y}} - D_{B} \frac{\partial }{\partial y}\left( {\frac{\partial C}{{\partial y}}} \right) - \frac{{D_{T} }}{{T_{\infty } }}\frac{\partial }{\partial y}\left( {\frac{\partial T}{{\partial y}}} \right) + k_{0}^{2} \left( {C - C_{\infty } } \right)\left( {\frac{T}{{T_{\infty } }}} \right)^{n} Exp\left[ {\frac{{E_{a} }}{\kappa T}} \right] = 0,$$

12 $$u\frac{\partial N}{{\partial x}} - D_{m} \left( {\frac{{\partial^{2} N}}{{\partial y^{2} }}} \right) + \frac{\partial N}{{\partial y}}v + \left[ {\frac{\partial }{\partial y}\left( {N\frac{\partial C}{{\partial y}}} \right)} \right]\frac{{bW_{c} }}{{\left( {C - C_{\infty } } \right)}} = 0,$$

where $\sigma$ is electrical conductivity and along axial and transverse direction the component form of velocity is (*u,v*). Now the governing Eqs. (10–11) and (15) turned the form as

13 $$S_{xx} - \mu \left[ {\frac{\partial u}{{\partial x}} - \frac{{n\delta^{2} }}{6}\left\{ {2\left( {\frac{\partial u}{{\partial x}}} \right)^{3} + \frac{\partial u}{{\partial x}}\left( {\frac{\partial u}{{\partial y}} + \frac{\partial v}{{\partial x}}} \right)^{2} + 2\frac{\partial u}{{\partial x}}\left( {\frac{\partial v}{{\partial y}}} \right)^{2} } \right\}} \right] = 0,$$

14 $$S_{xy} = S_{yx} = \frac{\mu }{2}\left[ {\left( {\frac{\partial v}{{\partial x}} + \frac{\partial u}{{\partial y}}} \right) - \frac{{n\delta^{2} }}{6}\left( {\frac{\partial v}{{\partial x}} + \frac{\partial u}{{\partial y}}} \right)\left\{ {2\left( {\frac{\partial u}{{\partial x}}} \right)^{2} + \left( {\frac{\partial v}{{\partial x}} + \frac{\partial u}{{\partial y}}} \right)^{2} + 2\left( {\frac{\partial v}{{\partial y}}} \right)^{2} } \right\}} \right],$$

15 $$S_{yy} - \mu \left[ {\frac{\partial v}{{\partial y}} - \frac{{n\delta^{2} }}{6}\left\{ {2\left( {\frac{\partial v}{{\partial y}}} \right)^{3} + \frac{\partial v}{{\partial y}}\left( {\frac{\partial u}{{\partial y}} + \frac{\partial v}{{\partial x}}} \right)^{2} + 2\frac{\partial v}{{\partial y}}\left( {\frac{\partial u}{{\partial x}}} \right)^{2} } \right\}} \right] = 0,$$

The orders of u, x, $\mu$, y, v, and $\delta$, are obtained using $\delta \sim O(\varepsilon ),u \sim O(1),x \sim O(1),v \sim O(\varepsilon ),y \sim O(\varepsilon ),\,and\,\mu \sim O(\varepsilon^{2} )$ and implementing order of magnitude analysis. By removing inertial effects and lower order terms from Eqs. (8–9), 2D Sutterby nano-fluid momentum equations turns the form as:

16 $$\begin{aligned} & \rho \left( {u\frac{\partial u}{{\partial x}} + v\frac{\partial u}{{\partial y}}} \right) + \frac{\partial p}{{\partial x}} - \frac{\mu }{2}\frac{{\partial^{2} u}}{{\partial y^{2} }}\left[ {1 - \frac{{m\delta^{2} }}{2}\left( {\frac{\partial u}{{\partial y}}} \right)^{2} } \right] + \sigma B_{0}^{2} u + g\left( {\frac{n + 1}{2}} \right) \\ & \left[ {\left( {T - T_{\infty } } \right)\left( {1 - C_{\infty } } \right)\beta \rho_{f} - \left( {\rho_{m} - \rho_{f} } \right)\left( {N - N_{\infty } } \right) - \left( {C - C_{\infty } } \right)\left( {\rho_{p} - \rho_{f} } \right)} \right] = 0, \\ \end{aligned}$$

17 $$\frac{\partial p}{{\partial y}} = 0,$$

By keeping in view Eq. (16) across the boundary layer no change in pressure will be occurred because pressure is function of *x* only. The buoyancy system developed for parabolic surface`s boundary layer is discussed in^44^ as:

18 $$- \frac{\partial p}{{\partial x}} - g\left( {\frac{n + 1}{2}} \right)\left[ {\left( {T - T_{\infty } } \right)\left( {1 - C_{\infty } } \right)\beta \rho_{f} - \left( {\rho_{m} - \rho_{f} } \right)\left( {N - N_{\infty } } \right) - \left( {C - C_{\infty } } \right)\left( {\rho_{p} - \rho_{f} } \right)} \right] = 0,$$

Momentum equation of Sutterby nano-fluid flow over a paraboloid surface is written as:

19 $$\begin{aligned} & \rho \left( {u\frac{\partial u}{{\partial x}} + v\frac{\partial u}{{\partial y}}} \right) + \frac{\partial p}{{\partial x}} - \frac{\mu }{2}\frac{{\partial^{2} u}}{{\partial y^{2} }}\left[ {1 - \frac{{m\delta^{2} }}{2}\left( {\frac{\partial u}{{\partial y}}} \right)^{2} } \right] + \sigma B_{0}^{2} u + g\left( {\frac{n + 1}{2}} \right) \\ & \left[ {\left( {T - T_{\infty } } \right)\left( {1 - C_{\infty } } \right)\beta \rho_{f} - \left( {\rho_{m} - \rho_{f} } \right)\left( {N - N_{\infty } } \right) - \left( {C - C_{\infty } } \right)\left( {\rho_{p} - \rho_{f} } \right)} \right] = 0, \\ \end{aligned}$$

The effect of catalytic surface on parabolic surface *A implies P* + Heat with rate = $$k_{0}$$$$\times$$ C $$\times$$ $$\exp \left( { - \frac{E}{RT}} \right)$$ = 0 by taking 1st order activation energy. Where, $$\beta$$ is thermal expansion, $$\beta^{ * }$$ is concentration expansion and $$g^{ * }$$ denotes gravitational acceleration.

From Ref.^45^ The set of suitable boundary conditions available from the bottom surface to the upper parabolic surface from where Sutterby nanofluid free stream flows is

20 $$u(\mathop{x}\limits^{\rightharpoonup} ,\overset{\lower0.5em\hbox{$\smash{\scriptscriptstyle\frown}$}}{A} \left( {\mathop{x}\limits^{\rightharpoonup} + e} \right)^{{\frac{1 - n}{2}}} ) = v(\mathop{x}\limits^{\rightharpoonup} ,\overset{\lower0.5em\hbox{$\smash{\scriptscriptstyle\frown}$}}{A} \left( {\mathop{x}\limits^{\rightharpoonup} + e} \right)^{{\frac{1 - n}{2}}} ) = 0,\,k\frac{\partial T}{{\partial y}} + Qk_{0} C\exp \left( { - \frac{E}{RT}} \right) = 0,\,$$

21 $$\begin{aligned} & \frac {D_{T}}{T_{\infty}} \frac{\partial T}{{\partial y}} + {T_{\infty}} \frac{\partial C}{{\partial y}} = 0, N=N_{w}\,\,at\,\,y = \overset{\lower0.5em\hbox{$\smash{\scriptscriptstyle\frown}$}}{A} \left( {x + e} \right)^{{\frac{1 - n}{2}}} , \\ & u(x,\infty ) \to U(x + e)^{n} ,\,\,C \to C_{\infty } ,\,T \to T_{\infty } ,\,N \to N_{\infty } \,\,as\,\,y \to \infty . \\ \end{aligned}$$

In view of above, activation energy is represented by *E*, gas constant is represented by *R*, $k_{0}$ is constant, reaction rate is denoted by *Q* and *P* is the product species.

Also from above reference, set of suitable similarity transformations to reduce the number of independent variables from governing equations and find the solution of equations and boundary conditions. Defined parameters are

22 $$\begin{aligned} u & = \frac{\partial \phi }{{\partial y}},\,v = \frac{\partial \phi }{{\partial x}},\,\phi (x,y) = f(\zeta )\sqrt {\frac{2\nu U}{{n + 1}}} (x + e)^{{\frac{n + 1}{2}}} ,\,\,\zeta = y\sqrt {\frac{n + 1}{2}} (x + e)^{{\frac{n - 1}{2}}} , \\ \theta & = \frac{{E(T - T_{\infty } )}}{{RT_{\infty }^{2} }},\,\phi = \frac{C}{{C_{\infty } }},\chi = \frac{N}{{N_{\infty } }}.\, \\ \end{aligned}$$

### Non-dimensional equations

Now the governing Eqs. (7–19) and (20–21) turned the form as:

23 $$\begin{aligned} & f\frac{{d^{2} f}}{{d\zeta^{2} }} - \left( {\frac{2n}{{n + 1}}} \right)\left( {\frac{df}{{d\zeta }}} \right)^{2} + \frac{1}{2}\frac{{d^{3} f}}{{d\zeta^{3} }}\left[ {1 - \frac{mDe}{2}\frac{n + 1}{2}\left( {\frac{{d^{2} f}}{{d\zeta^{2} }}} \right)^{2} } \right] + \lambda \left( {\theta - Nr\phi - Nc\chi } \right) \\ & \quad \quad - M\frac{df}{{d\zeta }} = 0, \\ \end{aligned}$$

24 $$\left( {1 + \frac{4}{3}Rd} \right)\frac{{d^{2} \theta }}{{d\zeta^{2} }} + \frac{1}{\Pr }\frac{{d^{2} \theta }}{{d\zeta^{2} }} + f\frac{d\theta }{{d\zeta }} + Nb\frac{d\theta }{{d\zeta }}\frac{d\phi }{{d\zeta }} + Nt\left( {\frac{d\theta }{{d\zeta }}} \right)^{2} = 0,$$

25 $$\frac{{d^{2} \phi }}{{d\zeta^{2} }} + Sc\left( {f\frac{d\phi }{{d\zeta }}} \right) + \frac{Nt}{{Nb}}\frac{{d^{2} \theta }}{{d\zeta^{2} }} = 0,$$

26 $$\frac{{d^{2} \chi }}{{d\zeta^{2} }} + Lb\left( {f\frac{d\chi }{{d\zeta }}} \right) - Pe\left( {\frac{d\chi }{{d\zeta }}\frac{d\phi }{{d\zeta }} + \frac{{d^{2} \phi }}{{d\zeta^{2} }}\left( {\varpi + \chi } \right)} \right) = 0,$$

### Dimensionless parameters

27 $$\left[ \begin{gathered} Sc = \frac{\upsilon }{{D_{B} }},\,Nt = \frac{{\tau D_{T} RT_{\infty } }}{E\upsilon },\,Nb = \frac{{\tau D_{B} C_{\infty } }}{\upsilon },\,De = \frac{{U^{3} (x + e)^{3n - 1} \delta^{2} }}{\upsilon },\, \hfill \\ \beta_{1} = \frac{{RT_{\infty } }}{E},M = \frac{{2\sigma B_{0}^{2} }}{{\rho U(n + 1)(x + e)^{n - 1} }},\hfill \\ \beta_{2} = \frac{{EQk_{0} C_{\infty } }}{{kRT_{\infty }^{2} (x + e)^{{\frac{n - 1}{2}}} }}\exp \left( { - \frac{E}{{RT_{\infty } }}} \right)\sqrt {\frac{2\upsilon }{{(n + 1)U}}} ,\,\Pr = \frac{{(\rho c_{p} )_{f} \nu }}{k}, \hfill \\ Nr = \frac{{g^{*} \beta RT_{\infty }^{2} }}{{EU^{2} (x + e)^{2n - 1} }},\,Nc = \frac{{g^{*} \beta^{*} C_{\infty } }}{{U^{2} (x + e)^{2n - 1} }}\,,Lb=\frac{{\nu}}{D_{m}},Pe=\frac{{bW_{c}}}{D_{m}},\varpi=\frac{N_{\infty}}{N_w-N_{\infty}} \hfill \\ \end{gathered} \right]$$

here *Sc* signifies Schmidt number,* Nt* is the thermophoresis parameter,* Nb* denotes Brownian motion, *De* shows Deborah number, $\beta_{1}$ nominate activation energy*,*$M$ denote the magnetic parameter, $\beta_{2}$ are reactant consumption parameters, *Pr* is Prandtl number, buoyancy parameter which depends on temperature is denoted by *Nr*, and buoyancy parameter which depends on concentration is denoted by $Nc$, *Lb* is the bioconvection Lewis number, Pe be the Pecklet number.

Consider the starting point of slot is $y = A\left( {x + e} \right)^{{\frac{1 - n}{2}}}$ because it is an accurate statement that for all values of *y* is zero on upper paraboloid surface. We know that starting point of slit is not the smallest rate of y and it is not a proper statement that arrangement of *y* = *0* in $\zeta$. All this done for making our equations dimensionless, $\zeta$ corresponds to the count of y whether it was smallest or highest which is defined as

$$\zeta = A\left( {\frac{n + 1}{2}} \right)^{\frac{1}{2}} \left( {\frac{U}{\upsilon }} \right)^{\frac{1}{2}} = \Omega$$

After this, boundary conditions are

28 $$\begin{aligned} & f(\Omega ) - \Omega \left( {\frac{1 - n}{{n + 1}}} \right) = 0,\,\frac{df(\Omega )}{{d\Omega }} = 0,\,\frac{d\theta (\Omega )}{{d\Omega }} + \beta_{2} \phi (\Omega )\exp \left( {\frac{\theta (\Omega )}{{1 + \beta_{1} \theta (\Omega )}}} \right) = 0, \\ & Nb\frac{d\phi (\Omega )}{{d\Omega }}+Nt\frac{d\theta (\Omega )}{{d\Omega }}= 0,\chi (\Omega )=1\,\,at\,\,\zeta = \Omega , \\ & \frac{df(\Omega )}{{d\Omega }} - 1 \to 0,\,\theta (\Omega ) \to 0,\,\,\phi (\Omega )\to 0,\chi (\Omega )\to 0\,\,\,as\,\,\,\,\Omega \to \infty . \\ \end{aligned}$$

Now define

29 $$\begin{aligned} f(\xi ) & = f(\zeta - \Omega ) = f(\zeta ),\,\,\theta (\xi ) = \theta (\zeta - \Omega ) = \theta (\zeta ),\,\, \\ \phi (\xi ) & = \phi (\zeta - \Omega ) = \phi (\zeta ),\chi (\xi ) = \chi (\zeta - \Omega ) = \chi (\zeta ). \\ \end{aligned}$$

for transformation of governing eq’s domain and b.c’s from $[\Omega ,\infty )\,\,to\,\,[0,\infty )$ and $\Omega$ is the horizontal paraboloid surface. The governing equations become

30 $$\begin{aligned} & f\frac{{d^{2} f}}{{d\xi^{2} }} - \left( {\frac{2n}{{n + 1}}} \right)\left( {\frac{df}{{d\xi }}} \right)^{2} + \frac{1}{2}\frac{{d^{3} f}}{{d\xi^{3} }}\left[ {1 - \frac{{\left( {mDe} \right)}}{2}\frac{{\left( {n + 1} \right)}}{2}\left( {\frac{{d^{2} f}}{{d\xi^{2} }}} \right)^{2} } \right] + \lambda \left( {\theta - Nr\phi - Nc\chi } \right) \\ & \quad \quad- M\frac{df}{{d\xi }} = 0, \\ \end{aligned}$$

31 $$\left( {1 + \frac{4}{3}Rd} \right)\frac{{d^{2} \theta }}{{d\xi^{2} }} + \frac{1}{\Pr }\frac{{d^{2} \theta }}{{d\xi^{2} }} + f\frac{d\theta }{{d\xi }} + Nb\frac{d\theta }{{d\xi }}\frac{d\phi }{{d\xi }} + Nt\left( {\frac{d\theta }{{d\xi }}} \right)^{2} = 0,$$

32 $$\frac{{d^{2} \phi }}{{d\xi^{2} }} + Sc\left( {f\frac{d\phi }{{d\xi }}} \right) + \frac{Nt}{{Nb}}\frac{{d^{2} \theta }}{{d\xi^{2} }} = 0,$$

33 $$\frac{{d^{2} \chi }}{{d\xi^{2} }} + Lb\left( {f\frac{d\chi }{{d\xi }}} \right) - Lb\left( {\frac{{\left( {1 - m} \right)}}{{\left( {1 + m} \right)}}\frac{df}{{d\xi }}\chi } \right) - Pe_{.} \left( {\frac{d\chi }{{d\xi }}\frac{d\phi }{{d\xi }} + \frac{{d^{2} \phi }}{{d\xi^{2} }}\left( {\varpi + \chi } \right)} \right) = 0,$$

With boundary constraints

34 $$\begin{aligned} & f(\xi ) - \Omega \left( {\frac{1 - n}{{n + 1}}} \right) = 0,\,\frac{df(\xi )}{{d\xi }} = 0,\,\frac{d\theta (\xi )}{{d\xi }} + \beta_{2} \phi (\xi )\exp \left( {\frac{\theta (\xi )}{{1 + \beta_{1} \theta (\xi )}}} \right) = 0, \\ & Nb\frac{d\phi (\xi )}{{d\xi }}+Nt\frac{d\theta (\xi )}{{d\xi }}= 0,\chi (\xi )=1,\,at\,\,\,\xi = 0, \\ \end{aligned}$$

35 $$\frac{df(\xi )}{{d\xi }} - 1 \to 0,\,\theta (\xi ) \to 0,\,\,\phi (\xi )\to 0,\,\chi (\xi )\to 0\,\,\,as\,\,\,\,\xi \to \infty .$$

## Numerical technique

In this section numerical technique is elaborated which used to solve our mathematical system. For numerical results of dimensionless ODE’s with boundary conditions, we used ‘bvp4c’ in built package of MATLAB. First step is to transform partial differential equation to ordinary differential equations by introducing dimension-less variables. Secondly for numerical solution, we took an initial guess and the domain of the solution by limiting our boundary conditions. Especially infinite condition to a finite one. Numerical system is

Let

36 $$\begin{aligned} f & = z_{1} ,f^{\prime } = z_{2} ,f^{\prime \prime } = z_{3} ,f^{\prime \prime \prime } = z_{3}^{\prime } , \\ \theta & = z_{4} ,\,\theta^{\prime } = z_{5} ,\,\theta^{\prime \prime } = z_{5}^{\prime } , \\ \phi & = z_{6} ,\,\phi^{\prime } = z_{7} ,\,\phi^{\prime \prime } = z_{7}^{\prime } , \\ \chi & = z_{8} ,\chi^{\prime } = z_{9} ,\chi^{\prime \prime } = z_{9}^{\prime } , \\ \end{aligned}$$

37 $$z_{3}^{\prime } = \frac{{2\left[ {\left( {\frac{2n}{{n + 1}}} \right)z_{2}^{2} - z_{1} z_{3} - \lambda \left( {\theta - Nr\phi - Nc\chi } \right) + M\,z_{2} } \right]}}{{\left[ {1 - \frac{{\left( {mDe} \right)}}{2}\frac{{\left( {n + 1} \right)}}{2}z_{3}^{2} } \right]}},$$

38 $$z_{5}^{\prime } = \frac{1}{{\left( {1 + \frac{4}{3}Rd + \frac{1}{\Pr }} \right)}} - z_{1} z_{5} - Nb\,z_{5} z_{7} - Nt\,z_{5}^{2} ,$$

39 $$z_{7}^{\prime } = - Sc\,z_{1} z_{7} - \frac{Nt}{{Nb}}z_{5}^{\prime } ,$$

40 $$z_{9}^{\prime } = Lb\left( {\left( {\frac{1 - m}{{1 + m}}} \right)z_{2} z_{8} - z_{1} z_{9} } \right) + Pe\left( {z_{9} z_{7} + z_{7}^{\prime } \left( {\varpi + \chi } \right)} \right),$$

With boundary conditions

41 $$z_{1} (\xi ) - \Omega \left( {\frac{1 - n}{{n + 1}}} \right) = 0,\,z_{2} (\xi ) = 0,\,z_{5} (\xi ) + \beta_{2} z_{6} (\xi )\exp \left( {\frac{{z_{4} (\xi )}}{{1 + \beta_{1} z_{4} (\xi )}}} \right) = 0,$$

42 $$Nbz_{7}+Ntz_{5}= 0,z_{8}=1,\,at\,\,\,\xi = 0,$$

43 $$z_{2} (\xi ) - 1 \to 0,\,z_{4} (\xi ) \to 0,\,\,z_{6} (\xi ) \to 0,\,\,z_{8} (\xi ) \to 0\,as\,\,\,\,\xi \to \infty .$$

## Discussion pertaining to deduced results

In this section the behavior of physical parameters discussed in detail on the basis of effects of these physical parameters on the different profiles. Also scrutinized the graphical significances of the physical flow parameters. During the flow of fluid, the influence of physical parameters on the flow profiles visualized graphically.

**Figure 3:**
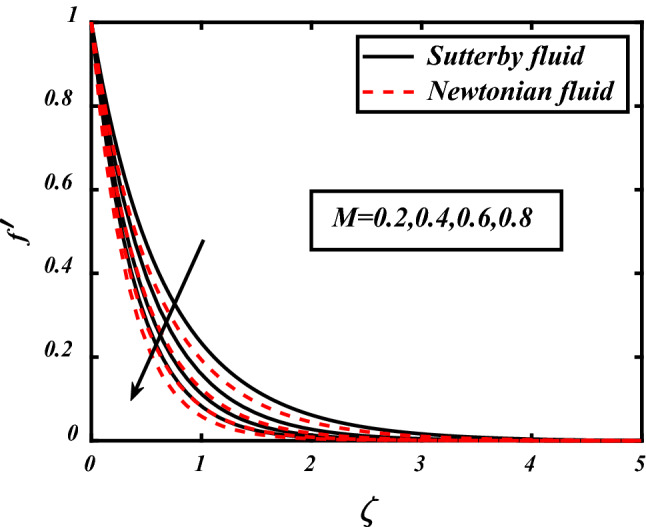
Expounds the impacts of $M$ on $f^{\prime }$.

**Figure 4:**
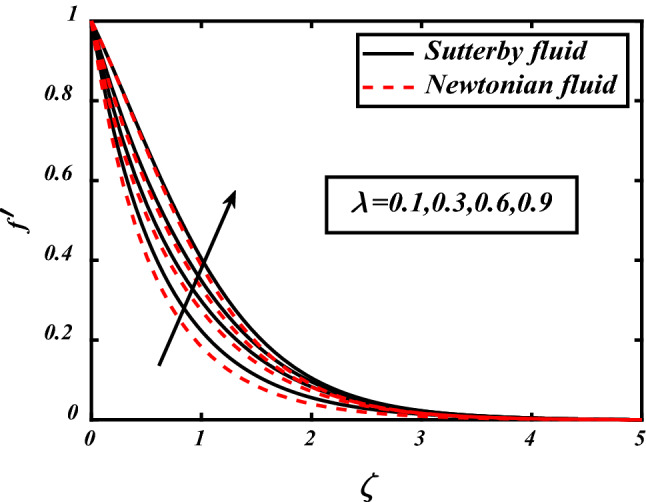
Expounds the impacts of $\lambda$ on $f^{\prime }$.

**Figure 5:**
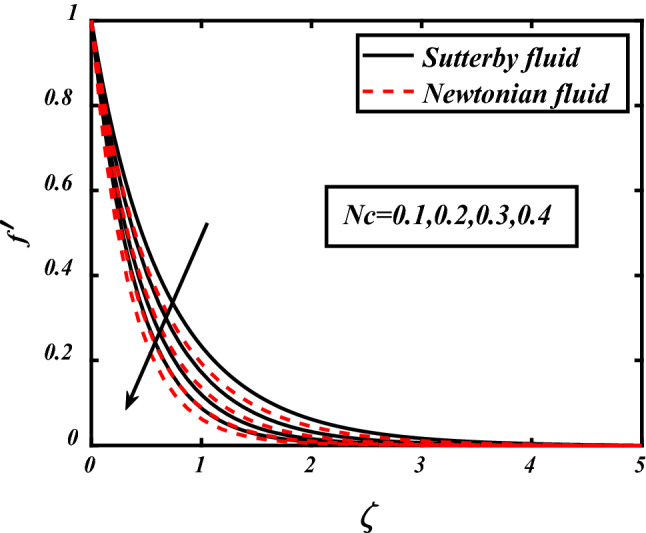
Expounds the impacts of $Nc$ on $f^{\prime }$.

**Figure 6:**
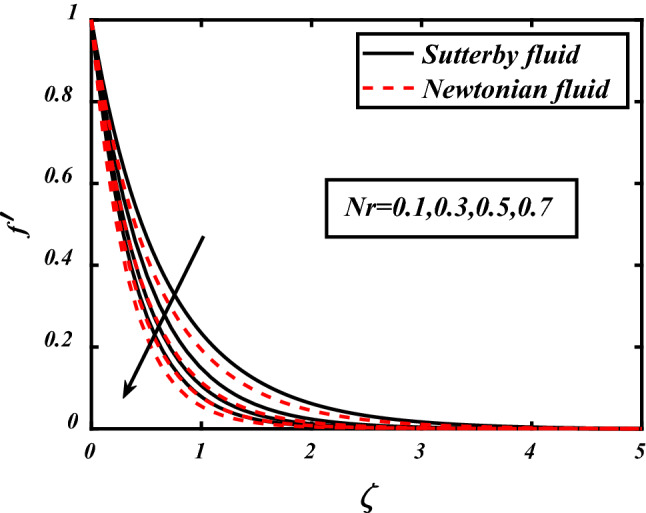
Expounds the impacts of $Nr$ on $f^{\prime }$.

In Figs. 3, 4, 5, 6, velocity profile of the problem is visualized graphically by considering various physical parameters and their effects on the profile. The thermal field is reduces with increasing the values of the Prandtl number. Physically thermal diffusivity of the fluid is decreased with increasing the values of the Prandtl number. A significant decrease in flow velocity is noted pertaining to the increase in magnetic field *M*, similarly buoyancy force *Nc* and Rayleigh number *Nr* also cause decrease in the flow whereas mixed convection parameter *⅄*, boosted the velocity of the flow. Physically buoyancy forces are increases with increment in the values of buoyancy ratio parameter.

**Figure 7:**
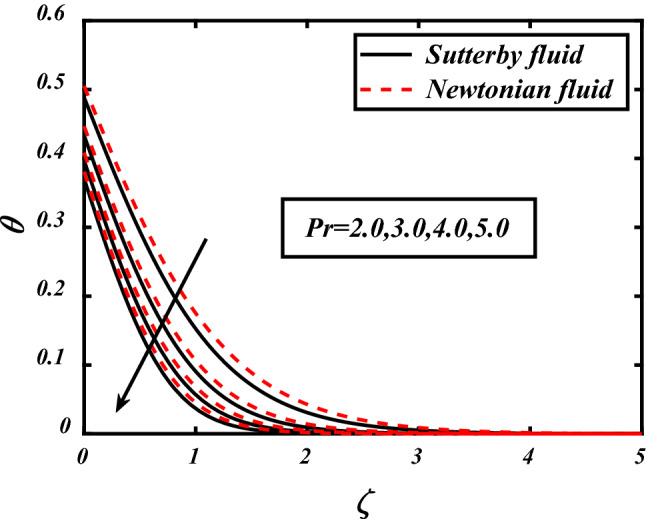
Expounds the impacts of $\Pr$ on $\theta$.

**Figure 8:**
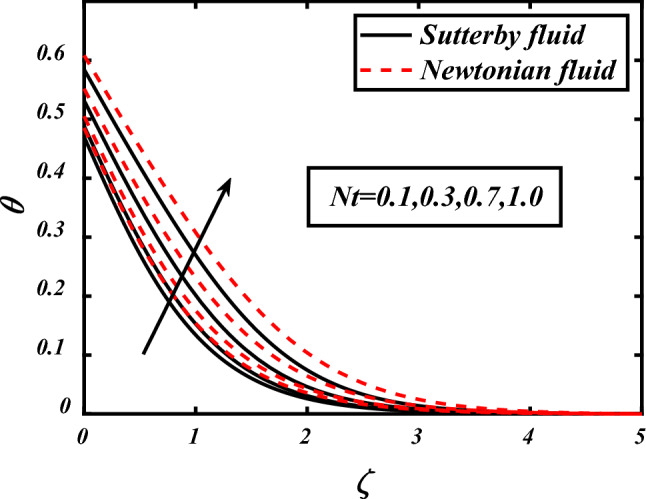
Expounds the impacts of $Nt$ on $\theta$.

**Figure 9:**
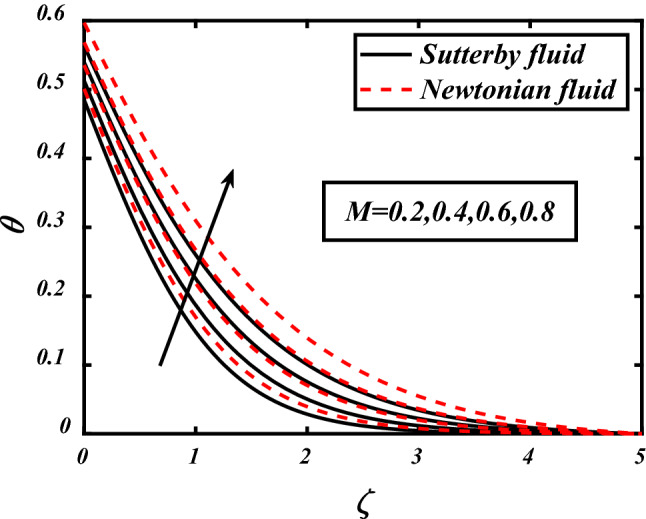
Expounds the impacts of $M$ on $\theta$.

**Figure 10:**
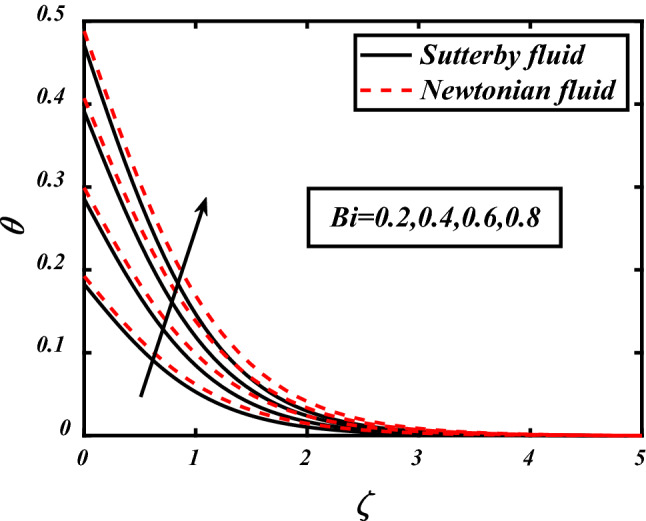
expounds the impacts of $Bi$ on $\theta$.

**Figure 11:**
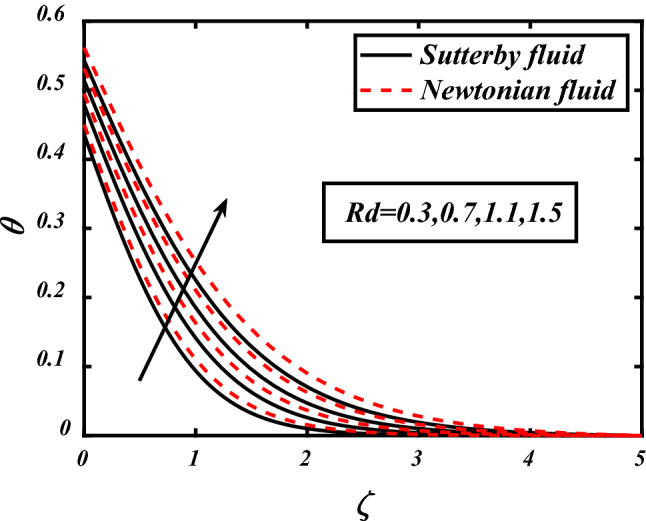
expounds the impacts of $Rd$ on $\theta$.

Figures 7, 8, 9, 10, 11 depicts the variations in the temperature profile of the flow and effects of important factors on those flow depends mainly. Prandtl number impacted badly on the temperature profile and causes decrease in flow whereas thermophoresis *Nt* which is force required to move particles from hot to cold region, magnetic field *M*, Biot number *Bi* which is ration of internal and boundary layer thermal resistance, Radiation parameter *Rd* boosted temperature and supports the flow of non-Newtonian nanofluid. Physically thermophoresis coefficient plays a manjor role in the heat transfer. Strong themoporesis forces are developed by increment in thermophoresis parameter which moves the nanoparticles from hot to cold region. Physical parameters matter highly in the flow of fluid; our mathematical models are developed for flow problems by involving these parameters restricted to some values. The thermal field is boosted via larger thermal Biot number. Physically, the heat conduction is enhanced with larger Biot number as result thermal field of species is boosted up.

**Figure 12:**
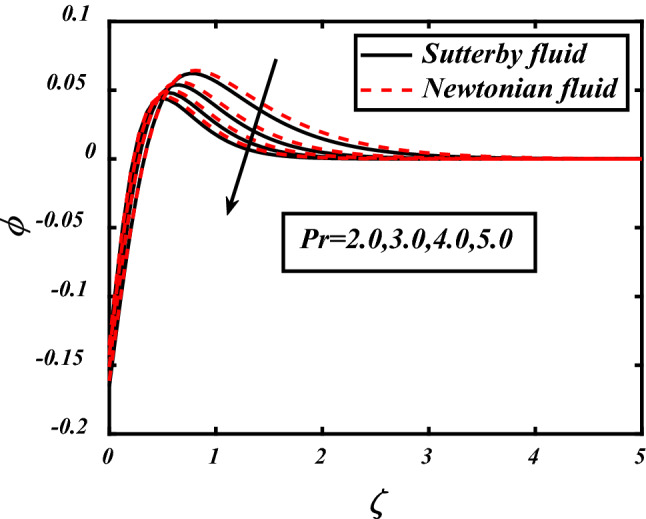
expounds the impacts of $\Pr$ on $\phi$.

**Figure 13:**
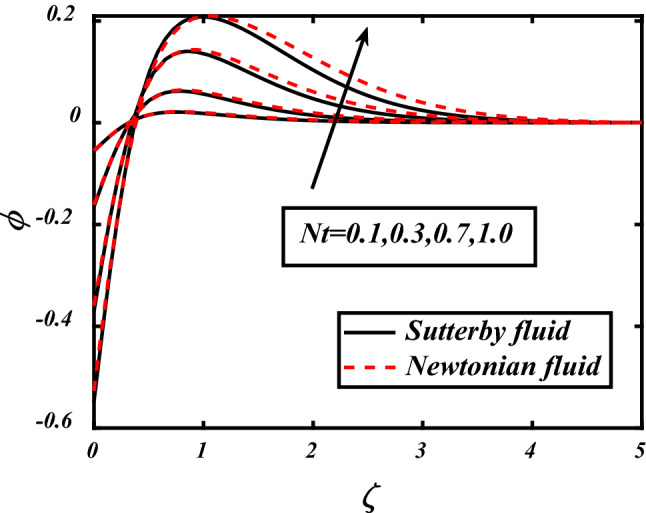
expounds the impacts of $Nt$ on $\phi$.

From the Figs. 12 and 13 significance of Prandtl number and thermophoresis effect on concentration profile is shown. Inverse of thermal diffusivity is Prandtl number, by taking higher values of *Pr* the temperature boundary layer in concentration profile reduced. Thermophoresis *Nt* moves the particles from higher to lower concentration, so by the increasing *Nt* Concentration profile shows huge change in movement of particles from higher towards lower concentration.

**Figure 14:**
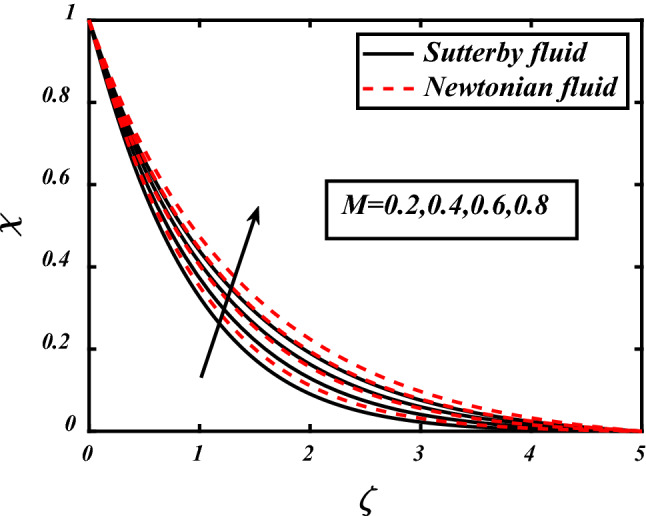
expounds the impacts of $M$ on $\chi$.

**Figure 15:**
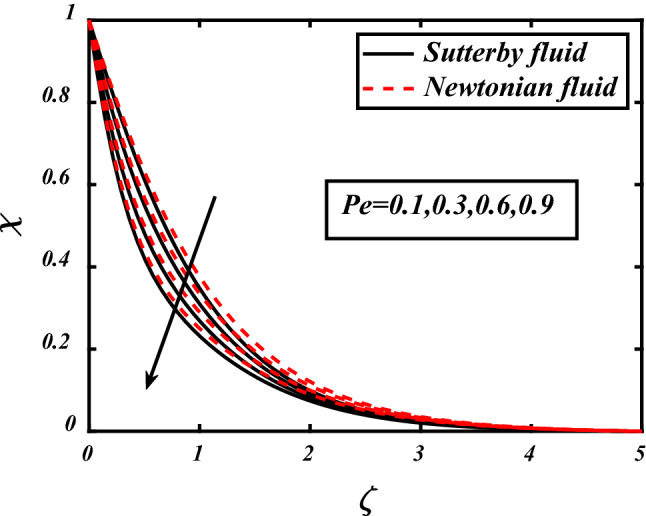
expounds the impacts of $Pe$ on $\chi$.

**Figure 16:**
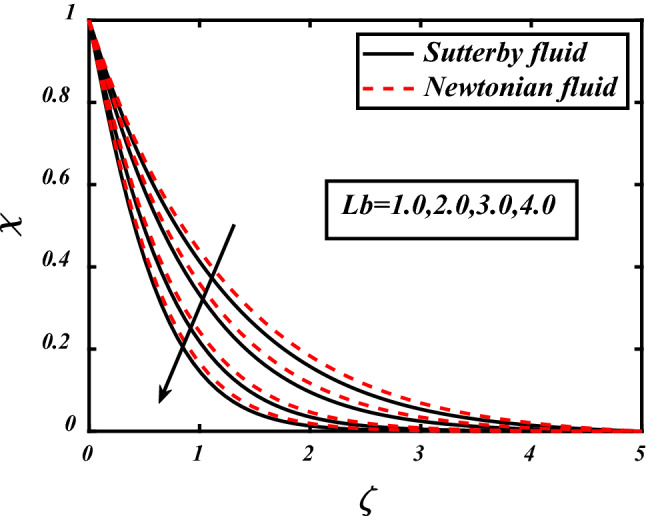
expounds the impacts of $Lb$ on $\chi$.

Influence in Microorganism profile due to different parameters depicted in Figs. 14, 15, 16. Clearly seen that by the increase of magnetic field parameter the movement of microorganisms also increased in both fluids whether it is non-Newtonian or Sutterby. The Peclet count *Pe* and bioconvection Lewis count *Lb* both have inverse effect then microorganism diffusivity, it is visualized in the above figures that concentration of microorganism decreased by the increase in *Lb* and *Pe*. Physically interpreted as the increase in *Lb* means decrease in motile microorganism due to this concentration profile also decreased. Similarly, same effect seen in case of Peclet number *Pe* additionally the motile microorganism thickness also lessens due to liquid particles movement because of *Pe*.

For numerical analysis, values of various parameters presented in tabular form and show the effects on profiles. Table 1 represented the significance of various parameters at the fixed values of *M* = 0.2,* ⅄* = 0.1,* Nc* = 0.1, and *Nr* = 0.1 on local skin friction coefficient. Profile is observed by giving various values to the parameters. Table 2 illustrated the significance of parameters at the fixed values *M* = 0.2,* Pr* = 5.0,* Nt* = 0.3,* Rd* = 0.8,* Bi* = 0.4,* ⅄* = 0.1, and 0.1 on local Nusselt count and comparison between the parameters are also observed. Table 3 visualized the significance of parameters at the fixed values of *Le* = 2.0,* Nb* = 0.2,* Pr* = 5.0,* Nt* = 0.3,* ⅄* = 0.1, and *Nc* = 0.1 on local Sherwood number and comparison between parameters also seen. From table values there is a decrease in numeric values reported by increasing the values of *Nb*,* Le*,* M*, and* Nc* on the local Sherwood number whereas by increasing the values of *Pr*,* Nt*, and *⅄* the rate of mass transport increases as showed in the values numerically. Basically Sherwood number is a dimensionless quantity which shows the ratio of convective to diffusive rate of mass transfer. Table 4 showed the impact of involving parameters on local microorganism density by giving fixed values to *M* = 0.2,* Lb* = 2.0,* Pe* = 0.1,* ⅄* = 0.1,* Nr* = 0.1,* and Nc* = 0.1*.* Local microorganism density number is the ration of motile microorganisms in the fluid flow from table we understand that with the increment in the *Lb, Pe,* and* ⅄* increment in the numeric values also noticed and density of microorganisms become more on the other hand by rising the values of *M*,* Nr*, and *Nc* downfall in numeric values captured. Table 5 demonstrated the comparative analysis of previous published results with current results and found that our results in good agreement with previous numerical values.

**Table 1:** Visualizes the significance of *M, ⅄, Nc,* and *Nr* on local skin friction coefficient.

*M*	$\lambda$	*Nc*	*Nr*	$- f^{\prime \prime } (0)$
**0.1**	0.1	0.1	0.1	**1.5796**
**0.3**				**1.6636**
**0.6**				**1.7809**
0.2	**0.3**	0.1	0.1	**1.6164**
	**0.6**			**1.6011**
	**0.9**			**1.5861**
0.2	0.1	**0.2**	0.1	**0.6292**
		**0.3**		**0.6317**
		**0.4**		**0.6393**
0.2	0.1	0.1	**0.2**	**1.6267**
			**0.3**	**1.6266**
			**0.4**	**1.6265**

Significant values are in [bold].

**Table 2:** Visualizes the significance of *M, Pr, Nt, Rd, Bi, ⅄,* and *Nc* on local Nusselt number.

*M*	*Pr*	*Nt*	*Rd*	*Bi*	$\lambda$	*Nc*	$- \theta^{\prime } (0)$
**0.1**	5.0	0.3	0.8	0.4	0.1	0.1	**0.4119**
**0.3**							**0.4066**
**0.6**							**0.3991**
0.2	**2.0**	0.3	0.8	0.4	0.1	0.1	**0.2576**
	**4.0**						**0.3763**
	**6.0**						**0.4340**
0.2	5.0	**0.2**	0.8	0.4	0.1	0.1	**0.4013**
		**0.4**					**0.3840**
		**0.6**					**0.3648**
0.2	5.0	0.3	**0.2**	0.4	0.1	0.1	**0.2960**
			**0.4**				**0.2881**
			**0.6**				**0.2808**
0.2	5.0	0.3	0.8	**0.2**	0.1	0.1	**0.1635**
				**0.5**			**0.3162**
				**0.8**			**0.4092**
0.2	5.0	0.3	0.8	0.4	**0.3**	0.1	**0.2743**
					**0.6**		**0.2747**
					**0.9**		**0.2750**
0.2	5.0	0.3	0.8	0.4	0.1	**0.2**	**0.2739**
						**0.3**	**0.2728**
						**0.4**	**0.2722**

Significant values are in [bold].

**Table 3:** Visualizes the significance of *Pr, Nb, Nt, Le, M, ⅄,* and *Nc* on local Sherwood number.

*Pr*	*Nb*	*Nt*	*Le*	*M*	$\lambda$	*Nc*	$- \phi^{\prime } (0)$
**2.0**	0.2	0.3	2.0	0.2	0.1	0.1	**0.3864**
**4.0**							**0.5644**
**6.0**							**0.6510**
5.0	**0.1**	0.3	2.0	0.2	0.1	0.1	**1.2277**
	**0.3**						**0.2455**
	**0.5**						**0.1535**
5.0	0.2	**0.2**	2.0	0.2	0.1	0.1	**0.8025**
		**0.4**					**1.1519**
		**0.6**					**1.4593**
5.0	0.2	0.3	**4.0**	0.2	0.1	0.1	**0.6065**
			**5.0**				**0.6043**
			**6.0**				**0.6026**
5.0	0.2	0.3	2.0	**0.1**	0.1	0.1	**0.6178**
				**0.3**			**0.6100**
				**0.6**			**0.5987**
5.0	0.2	0.3	2.0	0.2	**0.3**	0.1	**0.4114**
					**0.6**		**0.4120**
					**0.9**		**0.4126**
5.0	0.2	0.3	2.0	0.2	0.1	**0.2**	**0.4109**
						**0.3**	**0.4101**
						**0.4**	**0.3986**

Significant values are in [bold].

**Table 4:** Visualizes the significance *M, Lb, Pe, ⅄, Nr,* and *Nc* on local microorganism density number.

*M*	*Lb*	*Pe*	$\lambda$	*Nr*	*Nc*	$- \chi^{\prime } (0)$
**0.1**	2.0	0.1	0.1	0.1	0.1	**0.9806**
**0.3**						**0.9610**
**0.6**						**0.9343**
0.2	**3.0**	0.1	0.1	0.1	0.1	**1.1563**
	**4.0**					**1.3647**
	**5.0**					**1.5495**
0.2	2.0	**0.2**	0.1	0.1	0.1	**0.8749**
		**0.4**				**0.9513**
		**0.6**				**1.0281**
0.2	2.0	0.1	**0.3**	0.1	0.1	**0.9153**
			**0.6**			**0.9186**
			**0.9**			**0.9217**
0.2	2.0	0.1	0.1	**0.2**	0.1	**0.9130**
				**0.3**		**0.9129**
				**0.4**		**0.9127**
0.2	2.0	0.1	0.1	0.1	**0.2**	**0.9124**
					**0.3**	**0.9118**
					**0.4**	**0.9098**

Significant values are in [bold].

**Table 5:** Visualizes the comparison of current results with previous published results for the various values of *Pr*= 1.0, 2.0, 3.0, 5.0, 10.0.

_*Pr*_	Zeeshan et al. ^14^	Raju et al. ^46^	Pramanik ^47^	Current results
1.0	0.9548	0.9547341	0.95473	0.95475
2.0	1.4714	1.4714260	1.47142	1.47142
3.0	1.8692	1.8691341	1.86913	1.89915
5.0	2.5002	2.5001020	2.50010	2.50011
10.0	3.6603	3.6603121	3.66031	3.66030

## Conclusion

A novelty of this study is computed the numerical investigation of bioconvection effects involving thermal radiation, chemical process and activation energy on the flow of Sutterby nanofluid over a parabolic surface. During this investigation found that the physical parameters have larger impact on the movement of nanofluid and by giving variation to their values shows the prompt change in flow. There are some more results deduced from current study are:

- The fluid velocity enhanced due to the involvement of buoyancy forces.
- Temperature increment becomes possible with the elevation of thermal radiation, Brownian motion, thermophoresis and Biot number.
- Decline in concentration profile caused by the increase in Brownian motion and Prandtl number whereas elevation caused by *Nt*.
- The microorganisms profile is reduced via larger values of Peclet number and Lewis number.
- Heat transport enhanced due to the presence of motile microorganisms.
- By taking Sutterby nanofluid as a base fluid for flow over paraboloid surface is more suitable for heat and mass transport than non-Newtonian.

In future, researchers may work on this idea with various heat flux models like Fourier and Fick’s Law and also able to observe the behavior of hybrid nanofluid in this model.

## Data Availability

The datasets used and/or analyzed during the current study are available in the manuscript. Any additional information or data required available from corresponding author upon reasonable request.

## List of symbols

$$x,y$$: Cartesian coordinates $$\left( - \right)$$
$k$: Nanofluid Thermal conductivity (W/mK)
$q_{r}$: Radiative heat flux (W/m^2^)
$B_{0}$: Magnetic parameter (Tesla)
$$Sc$$: Schmidht number $$\left( - \right)$$
$$k_{1}$$: Chemical reaction $$\left( - \right)$$
$$k_{0}$$: Gas constant $$\left( - \right)$$
$$R$$: Reaction constant $$\left( - \right)$$
$$Bi$$: Biot number $$\left( - \right)$$
$$M$$: Magnetic strength $$\left( - \right)$$
$\left( {\rho C_{p} } \right)_{nf}$: Heat capacity of nanofluid (J/kg·K)
$D_{m} \,$: Microorganism’s diffusion coefficient $\left( - \right)$
$E$: Activation energy variable $\left( - \right)$
$$u,v$$: Velocity components (m/s)
$D_{B}$: Brownian motion coefficient (m^2^/s)
$$D_{T}$$: Thermophoresis coefficient (m^2^/s)
$$g^{*}$$: Gravitational acceleration $$\left( - \right)$$
$$\Pr$$: Prandtl number $$\left( - \right)$$
$C$: Concentration of particles (mol/m^3^)
$C_{\infty }$: Free stream concentration (mol/m^3^)
$C_{w}$: Surface concentration (mol/m^3^)
$T$: Temperature of particles (K)
$$T_{\infty }$$: Ambient temperature (T)
$$T_{w}$$: Surface temperature (T)
$$N$$: Microorganism’s density $$\left( - \right)$$
$$N_{\infty }$$: Ambient microorganisms $$\left( - \right)$$
$$N_{w}$$: Surface microorganisms $$\left( - \right)$$
$C_{f}$: Skin friction parameter $\left( - \right)$
$$Nt$$: Thermophoresis parameter $$\left( - \right)$$
$Nb$: Brownian motion parameter $\left( - \right)$
$Nr$: Buoyancy ratio parameter $\left( - \right)$
$Nc$: Bioconvection Rayleigh number $\left( - \right)$
$$Rd$$: Thermal radiation $$\left( - \right)$$
$$Pe$$: Peclet number $$\left( - \right)$$
$Le$: Bioconvection Lewis number $\left( - \right)$

## Greek symbols

$\rho_{p}$: Nanoparticle density (kg/m^3^)
$\tau$: Ratio of heat capacitance $\left( - \right)$
$$\alpha$$: Thermal diffusivity (m^2^/s)
$\delta$: Thermal ratio parameter $\left( - \right)$
$$\upsilon$$: Kinematic viscosity (m^2^/s)
$\mu$: Dynamic viscosity (kg m^−1^ s^−1^)
$\lambda$: Mixed convection parameter $\left( - \right)$
$$\gamma^{**}$$: Volume fraction of microorganisms $$\left( - \right)$$
$$\beta^{*}$$: Volume suspension $$\left( - \right)$$
$\rho_{f}$: Nanofluid density (kg/m^3^)
$\sigma$: Stefan–Boltzman constant $\left( - \right)$
$\sigma^{*}$: Electric conductivity of fluid $\left( - \right)$
$\varpi$: Microorganisms difference variable

## Subscripts

$$f$$: Fluid
$$p$$: Particle

## References

[1] Choi, S. U. & Eastman, J. A. (1995). *Enhancing thermal conductivity of fluids with nanoparticles* (No. ANL/MSD/CP-84938; CONF-951135–29). Argonne National Lab. (ANL), Argonne, IL (United States).

[2] Sabir Z, Imran A, Umar M, Zeb M, Shoaib M, Raja MAZ (2021). A numerical approach for 2-D Sutterby fluid-flow bounded at a stagnation point with an inclined magnetic field and thermal radiation impacts. Therm. Sci..

[3] Madhukesh JK, Ramesh GK, Prasannakumara BC, Shehzad SA, Abbasi FM (2021). Bio-Marangoni convection flow of Casson nanoliquid through a porous medium in the presence of chemically reactive activation energy. Appl. Math. Mech..

[4] Song YQ, Waqas H, Al-Khaled K, Farooq U, Khan SU, Khan MI, Qayyum S (2021). Bioconvection analysis for Sutterby nanofluid over an axially stretched cylinder with melting heat transfer and variable thermal features: A Marangoni and solutal model. Alex. Eng. J..

[5] Waqas H, Farooq U, Muhammad T, Hussain S, Khan I (2021). Thermal effect on bioconvection flow of Sutterby nanofluid between two rotating disks with motile microorganisms. Case Stud. Therm. Eng..

[6] Ramesh GK, Madhukesh JK (2021). Activation energy process in hybrid CNTs and induced magnetic slip flow with heat source/sink. Chin. J. Phys..

[7] Usman AH, Saeed Khan N, Ali Rano S, Maitama M (2021). Study of heat and mass transfer in MHD flow of sutterby nanofluid over a curved stretching sheet with magnetic dipole and effect. Thai J. Math..

[8] Mabood F, Mackolil J, Mahanthesh BSEP, Rauf A, Shehzad SA (2021). Dynamics of Sutterby fluid flow due to a spinning stretching disk with non-Fourier/Fick heat and mass flux models. Appl. Math. Mech..

[9] Sajid T, Jamshed W, Shahzad F, Akgül EK, Nisar KS, Eid MR (2022). Impact of gold nanoparticles along with Maxwell velocity and Smoluchowski temperature slip boundary conditions on fluid flow: Sutterby model. Chin. J. Phys..

[10] Aldabesh A, Haredy A, Al-Khaled K, Khan SU, Tlili I (2022). Darcy resistance flow of Sutterby nanofluid with microorganisms with applications of nano-biofuel cells. Sci. Rep..

[11] Bouslimi J, Alkathiri AA, Althagafi TM, Jamshed W, Eid MR (2022). Thermal properties, flow and comparison between Cu and Ag nanoparticles suspended in sodium alginate as Sutterby nanofluids in solar collector. Case Stud. Therm. Eng..

[12] Faizan M, Ali F, Loganathan K, Zaib A, Reddy CA, Abdelsalam SI (2022). Entropy analysis of sutterby nanofluid flow over a Riga sheet with gyrotactic microorganisms and Cattaneo–Christov double diffusion. Mathematics.

[13] Shah Z, Kumam P, Deebani W (2020). Radiative MHD Casson Nanofluid flow with activation energy and chemical reaction over past nonlinearly stretching surface through Entropy generation. Sci. Rep..

[14] Zeeshan A, Awais M, Alzahrani F, Shehzad N (2021). Energy analysis of non-Newtonian nanofluid flow over parabola of revolution on the horizontal surface with catalytic chemical reaction. Heat Transf..

[15] Punith Gowda RJ, Naveen Kumar R, Jyothi AM, Prasannakumara BC, Sarris IE (2021). Impact of binary chemical reaction and activation energy on heat and mass transfer of marangoni driven boundary layer flow of a non-Newtonian nanofluid. Processes.

[16] Abdal S, Siddique I, Alshomrani AS, Jarad F, Din ISU, Afzal S (2021). Significance of chemical reaction with activation energy for Riga wedge flow of tangent hyperbolic nanofluid in existence of heat source. Case Stud. Therm. Eng..

[17] Haq F, Saleem M, Khan MI (2021). Investigation of mixed convection magnetized Casson nanomaterial flow with activation energy and gyrotactic microorganisms. J. Phys. Commun..

[18] Venkateswarlu A, Suneetha S, Babu MJ, Kumar JG, Raju CSK, Al-Mdallal Q (2021). Significance of magnetic field and chemical reaction on the natural convective flow of hybrid nanofluid by a sphere with viscous dissipation: A statistical approach. Nonlinear Eng..

[19] Benhanifia K, Redouane F, Lakhdar R, Brahim M, Al-Farhany K, Jamshed W, Raizah Z (2022). Investigation of mixing viscoplastic fluid with a modified anchor impeller inside a cylindrical stirred vessel using Casson–Papanastasiou model. Sci. Rep..

[20] Haq I, Bilal M, Ahammad NA, Ghoneim ME, Ali A, Weera W (2022). Mixed convection nanofluid flow with heat source and chemical reaction over an inclined irregular surface. ACS Omega.

[21] Jamshed W, Eid MR, Al-Hossainy AF, Raizah Z, Tag El Din ESM, Sajid T (2022). Experimental and TDDFT materials simulation of thermal characteristics and entropy optimized of Williamson Cu-methanol and Al_2_O_3_-methanol nanofluid flowing through solar collector. Sci. Rep..

[22] Ali B, Hussain S, Nie Y, Ali L, Hassan SU (2020). Finite element simulation of bioconvection and Cattaneo–Christov effects on micropolar based nanofluid flow over a vertically stretching sheet. Chin. J. Phys..

[23] Waqas H, Khan SA, Khan SU, Khan MI, Kadry S, Chu YM (2021). Falkner–Skan time-dependent bioconvrction flow of cross nanofluid with nonlinear thermal radiation, activation energy and melting process. Int. Commun. Heat Mass Transf..

[24] Khan SA, Waqas H, Naqvi SMRS, Alghamdi M, Al-Mdallal Q (2021). Cattaneo–Christov double diffusions theories with bio-convection in nanofluid flow to enhance the efficiency of nanoparticles diffusion. Case Stud. Therm. Eng..

[25] Imran M, Farooq U, Muhammad T, Khan SU, Waqas H (2021). Bioconvection transport of Carreau nanofluid with magnetic dipole and nonlinear thermal radiation. Case Stud. Therm. Eng..

[26] Hayat T, Ullah I, Muhammad K, Alsaedi A (2021). Gyrotactic microorganism and bio-convection during flow of Prandtl–Eyring nanomaterial. Nonlinear Eng..

[27] El Din SM, Darvesh A, Ayub A, Sajid T, Jamshed W, Eid MR, Dapozzo CLA (2022). Quadratic multiple regression model and spectral relaxation approach for carreau nanofluid inclined magnetized dipole along stagnation point geometry. Sci. Rep..

[28] Asjad MI, Zahid M, Jarad F, Alshari AM (2022). Bioconvection flow of MHD viscous nanofluid in the presence of chemical reaction and activation energy. Math. Probl. Eng..

[29] Rehman MIU, Chen H, Hamid A, Qayyum S, Jamshed W, Raizah Z, Din ESMTE (2022). Soret and Dufour influences on forced convection of Cross radiative nanofluid flowing via a thin movable needle. Sci. Rep..

[30] Wang F, Ahmad S, Al Mdallal Q, Alammari M, Khan MN, Rehman A (2022). Natural bio-convective flow of Maxwell nanofluid over an exponentially stretching surface with slip effect and convective boundary condition. Sci. Rep..

[31] Madhukesh JK, Ramesh GK, Aly EH, Chamkha AJ (2022). Dynamics of water conveying SWCNT nanoparticles and swimming microorganisms over a Riga plate subject to heat source/sink. Alex. Eng. J..

[32] Imran M, Kamran T, Khan SA, Muhammad T, Waqas H (2022). Physical attributes of bio-convection in nanofluid flow through a paraboloid of revolution on horizontal surface with motile microorganisms. Int. Commun. Heat Mass Transf..

[33] Shahzad F, Jamshed W, Eid MR, Safdar R, Putri Mohamed Isa SS, El Din SM, Iqbal A (2022). Thermal cooling efficacy of a solar water pump using Oldroyd-B (aluminum alloy-titanium alloy/engine oil) hybrid nanofluid by applying new version for the model of Buongiorno. Sci. Rep..

[34] Awan AU, Shah SAA, Ali B (2022). Bio-convection effects on Williamson nanofluid flow with exponential heat source and motile microorganism over a stretching sheet. Chin. J. Phys..

[35] Ramesh GK, Madhukesh JK, Aly EH, Pop I (2022). Modified Buongiorno’s model for biomagnetic hybrid nanoliquid past a permeable moving thin needle. Int. J. Numer. Methods Heat Fluid Flow.

[36] Daniel YS, Aziz ZA, Ismail Z, Salah F (2019). Thermal radiation on unsteady electrical MHD flow of nanofluid over stretching sheet with chemical reaction. J. King Saud Univ.-Sci..

[37] Sobamowo MG (2018). Combined effects of thermal radiation and nanoparticles on free convection flow and heat transfer of casson fluid over a vertical plate. Int. J. Chem. Eng..

[38] Venkata KB, Jayaramireddy K, Charankumar G (2019). MHD and thermal radiation effects of a nanofluid over a stretching sheet using HAM. Int. J. Recent Technol. Eng. (IJRTE).

[39] Shahzad F, Bouslimi J, Gouadria S, Jamshed W, Eid MR, Safdar R, Nisar KS (2022). Hydrogen energy storage optimization in solar-HVAC using Sutterby nanofluid via Koo–Kleinstreuer and Li (KKL) correlations model: A solar thermal application. Int. J. Hydrog. Energy.

[40] Dash RK, Mishra SR, Pattnaik PK (2022). Influence of radiative heat energy on the MHD flow of Cu-kerosene nanofluid over a vertical plate: Laplace transform technique. Biointerface Res. Appl. Chem..

[41] Rehman MIU, Chen H, Hamid A, Jamshed W, Eid MR, El Din SM, Abd-Elmonem A (2023). Effect of Cattaneo–Christov heat flux case on Darcy–Forchheimer flowing of Sutterby nanofluid with chemical reactive and thermal radiative impacts. Case Stud. Therm. Eng..

[42] Hayat T, Zahir H, Mustafa M, Alsaedi A (2016). Peristaltic flow of Sutterby fluid in a vertical channel with radiative heat transfer and compliant walls: A numerical study. Res. Phys..

[43] Salahuddin T, Ali Z, Awais M, Khan M, Altanji M (2022). A flow behavior of Sutterby nanofluid near the catalytic parabolic surface. Int. Commun. Heat Mass Transf..

[44] Makinde OD, Animasaun IL (2016). Bioconvection in MHD nanofluid flow with nonlinear thermal radiation and quartic autocatalysis chemical reaction past an upper surface of a paraboloid of revolution. Int. J. Therm. Sci..

[45] Chaudhary MA, Merkin JH (1994). Free-convection stagnation-point boundary layers driven by catalytic surface reactions: I the steady states. J. Eng. Math..

[46] Raju CSK, Sandeep N, Sugunamma V, Babu MJ, Reddy JR (2016). Heat and mass transfer in magnetohydrodynamic Casson fluid over an exponentially permeable stretching surface. Eng. Sci. Technol. Int. J..

[47] Pramanik S (2014). Casson fluid flow and heat transfer past an exponentially porous stretching surface in presence of thermal radiation. Ain shams Eng. J..

